# Supplementary material for: Photodynamic Inactivation of Foodborne Bacteria: Screening of 32 Potential Photosensitizers
Source: Foods. 2024 Jan 31;13(3):453. doi: 10.3390/foods13030453 (PMC10855769; doi:10.3390/foods13030453)
Supplement: Supplementary file 1 [file foods-13-00453-s001.zip › foods-2821397-supplementary.pdf]

MB

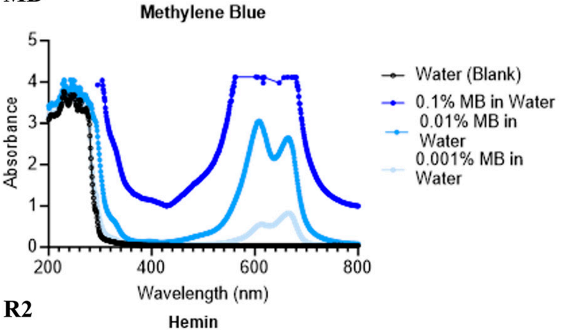

R1

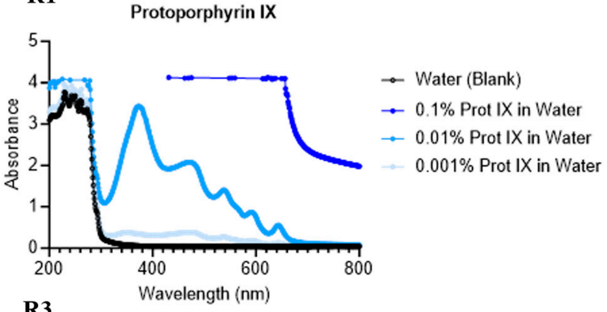

R2

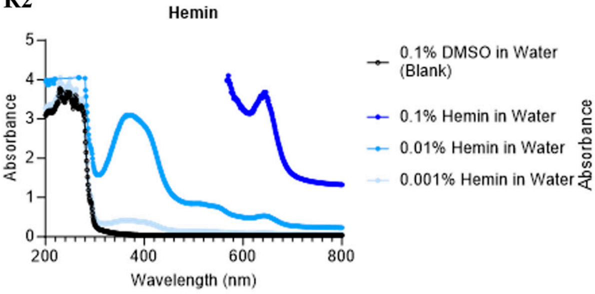

R3

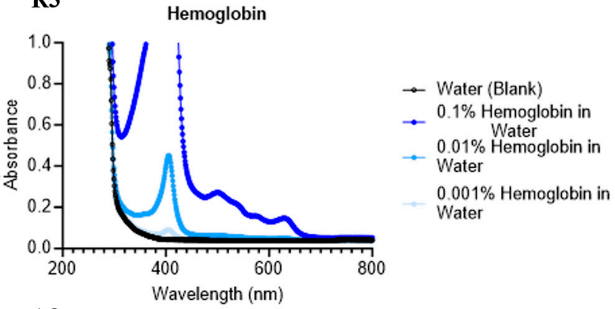

A2

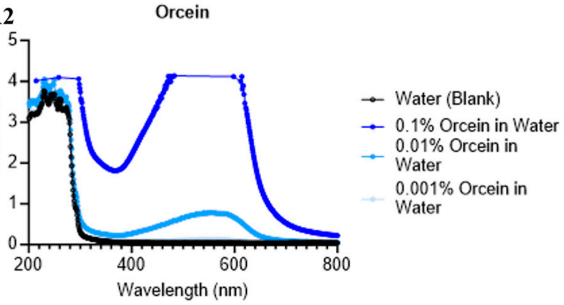

A3

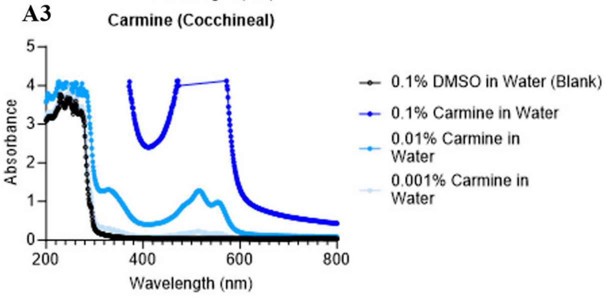

G1

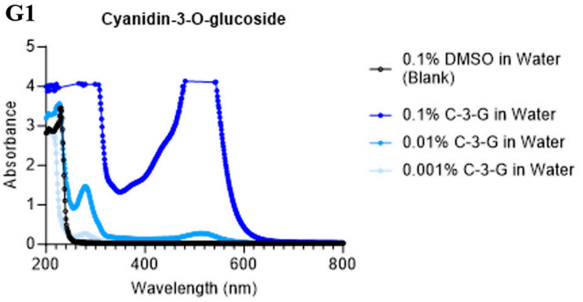

G2

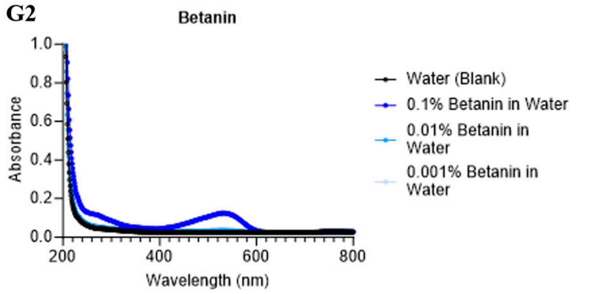

G3

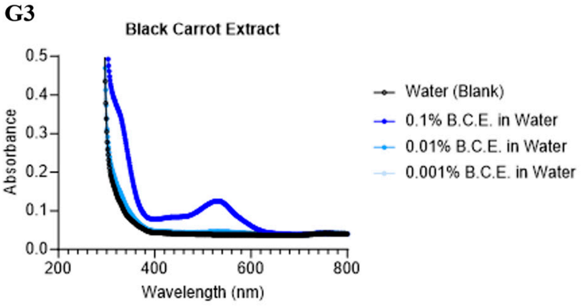

G4

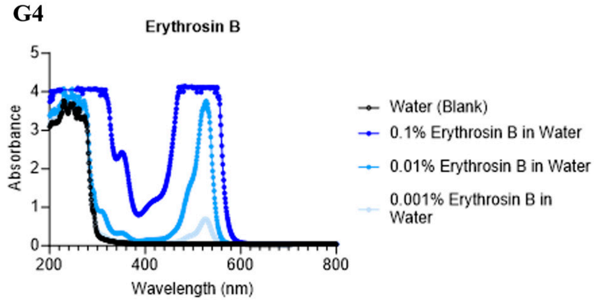

G5

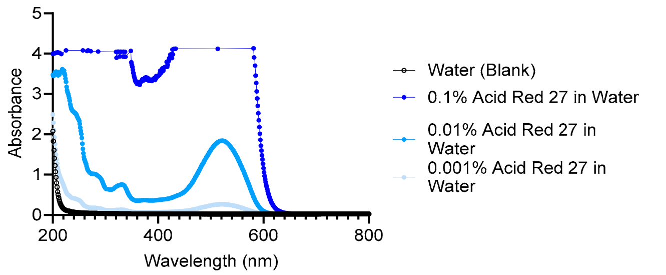

G6

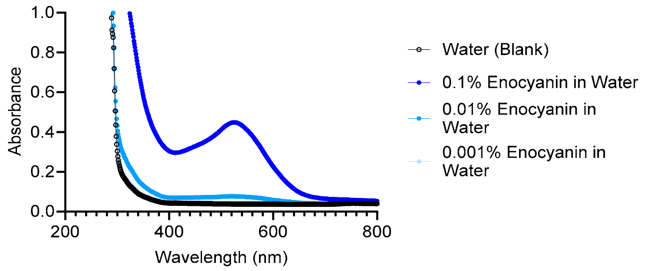

G7

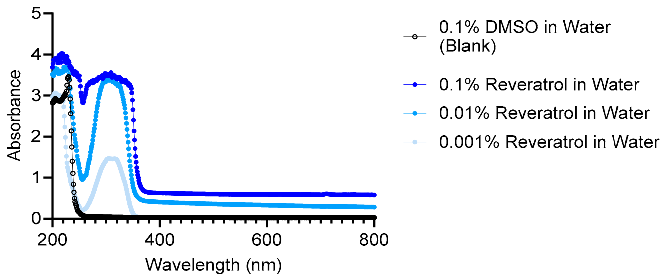

C1

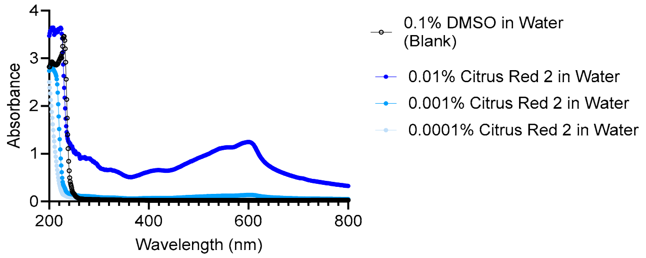

C2

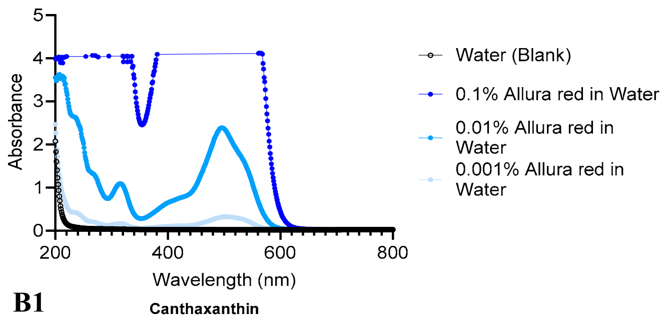

C3

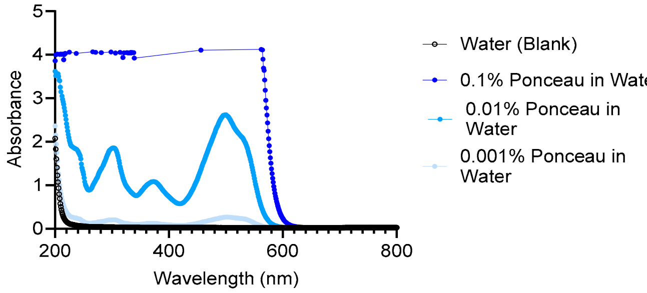

B1

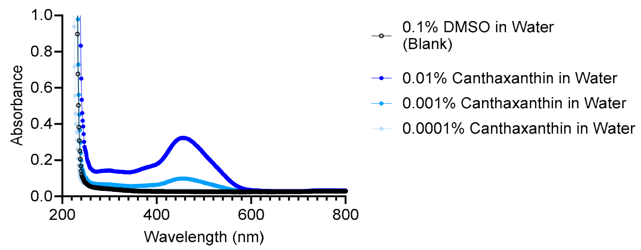

B2

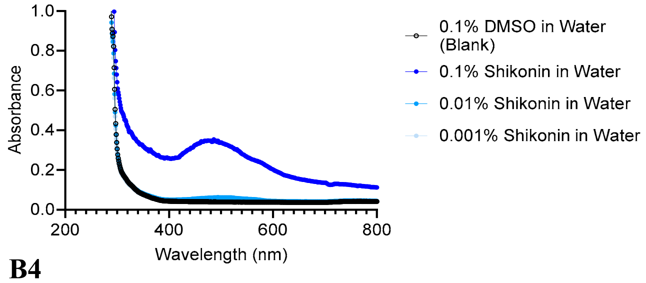

B3

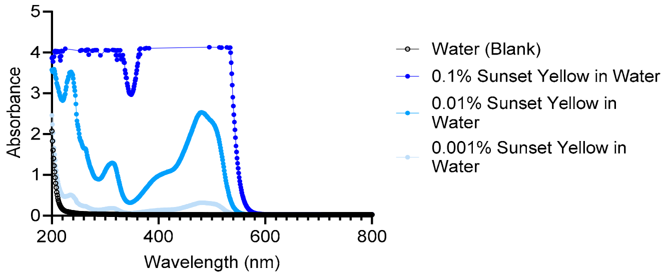

B4

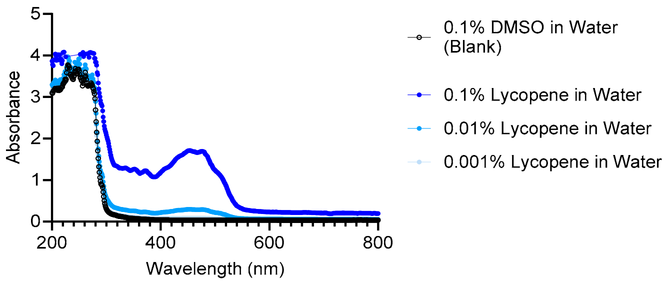

B5

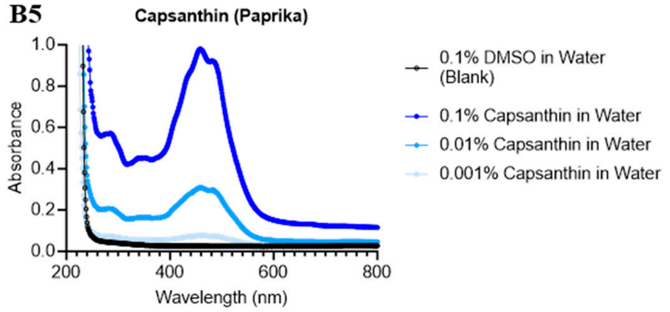

B6

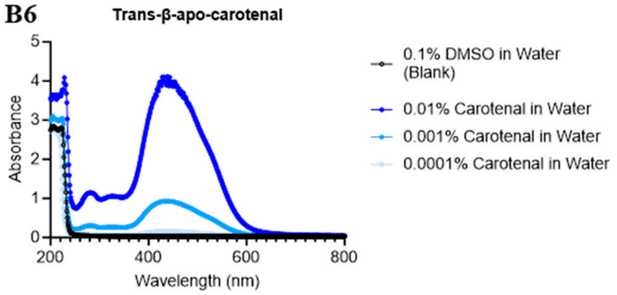

B7

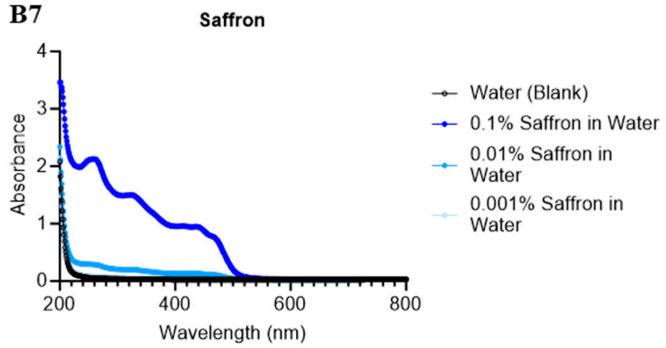

I1

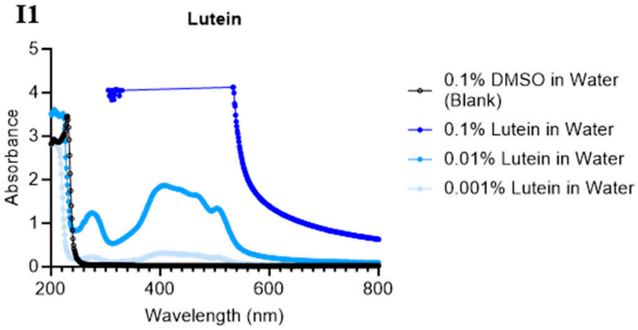

I2

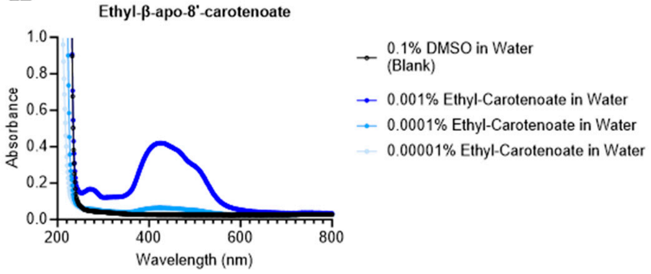

I3

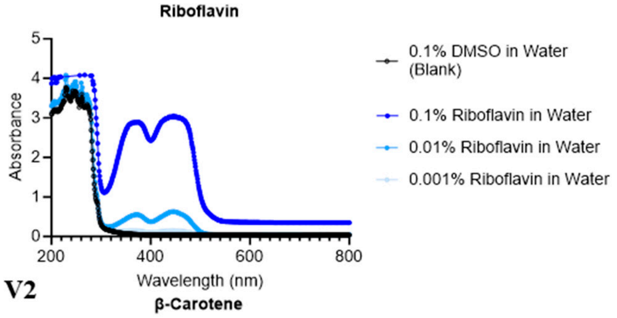

V1

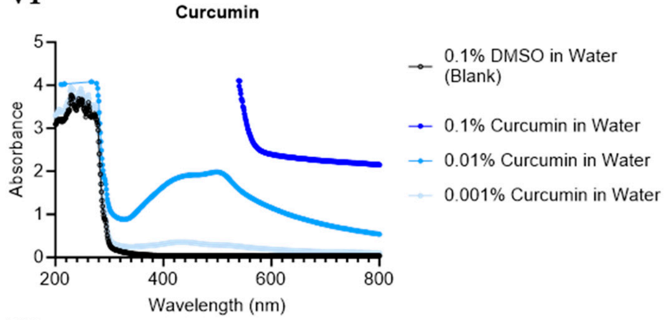

V2

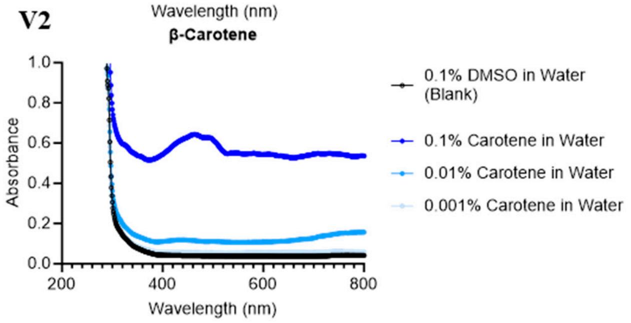

V3

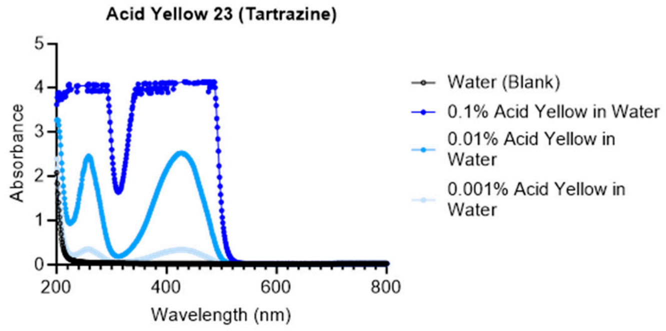

V4

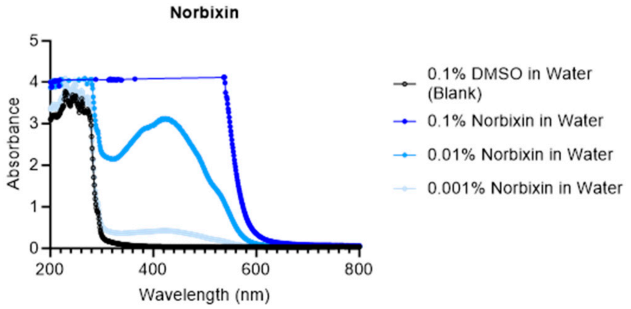

V5

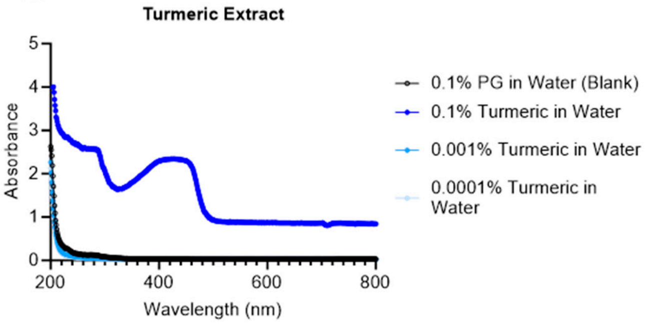

UV1

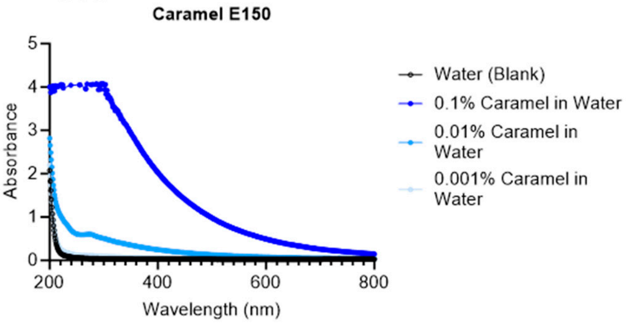

Figure S1. Spectral scans of photosensitizer candidates used in this study. The absorbance was measured over the wavelength range of 200 – 800 nm using a UV-Vis spectrophotometer. Here, **MB** corresponds to Methylene Blue, **R** corresponds to the photosensitizers used with red wavelength (630 nm), **A** corresponds to the photosensitizers used with amber wavelength (590 nm), **G** corresponds to the photosensitizers used with green wavelength (527 nm), **C** corresponds to the photosensitizers used with cyan wavelength (505 nm), **B** corresponds to the photosensitizers used with blue wavelength (470 nm), **I** corresponds to the photosensitizers used with indigo wavelength (445 nm), **V** corresponds to the photosensitizers used with violet wavelength (420 nm), and **UV** corresponds to the photosensitizers used with ultraviolet wavelength (365 nm). Titanium dioxide was not tested as it formed insoluble crystals and was used as a suspension in water instead.

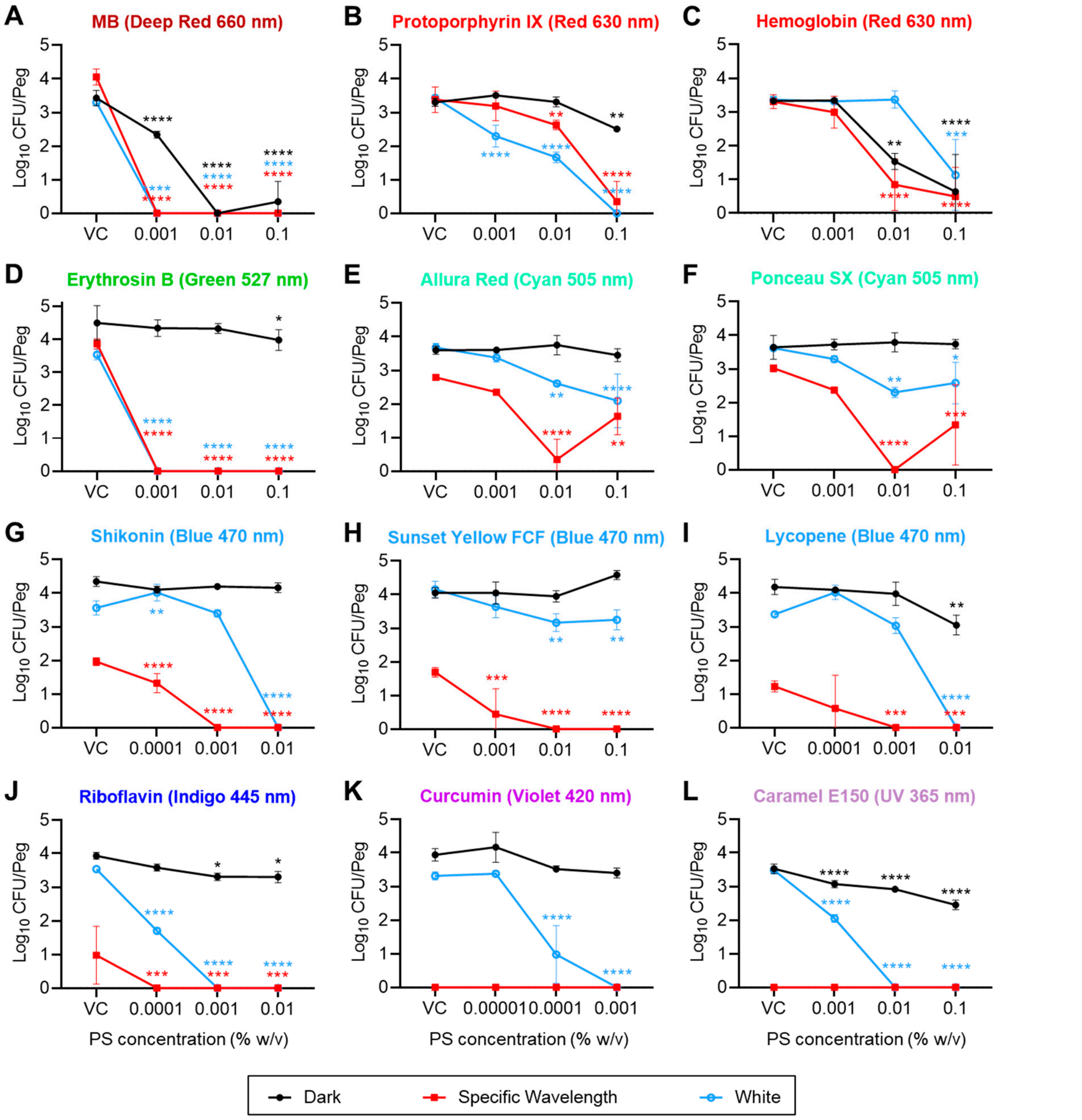

Figure S2. Recovery of viable *Pseudomonas fragi* ATCC 4973 from MBEC pegs (Log_{10} CFU/peg). Biofilms were challenged with methylene blue **(A)**, protoporphyrin IX **(B)**, hemoglobin **(C)**, erythrosin B **(D)**, allura red **(E)**, ponceau SX **(F)**, shikonin **(G)**, sunset yellow FCF **(H)**, tomato lycopene extract **(I)**, riboflavin **(J)**, curcumin **(K)**, or caramel color E150 **(L)**, at the indicated concentrations (x-axis; VC = Vehicle Controls), and subjected to the indicated irradiation conditions: dark (represented by black lines) *vs* specific wavelength (monochromatic light indicated above each graph, represented by red lines) *vs* white light (polychromatic light represented by blue lines). Symbols and error bars represent the mean ± standard deviation for 3 replicate pegs. Statistical significance, as indicated for certain data points, was evaluated by 2-way ANOVA and *p*-values (relative to the corresponding "VC" data point for each irradiation condition) were corrected for multiple comparisons using Dunnett's method. *, $p<0.05$; **, $p<0.01$; ***, $p<0.001$; ****, $p<0.0001$.

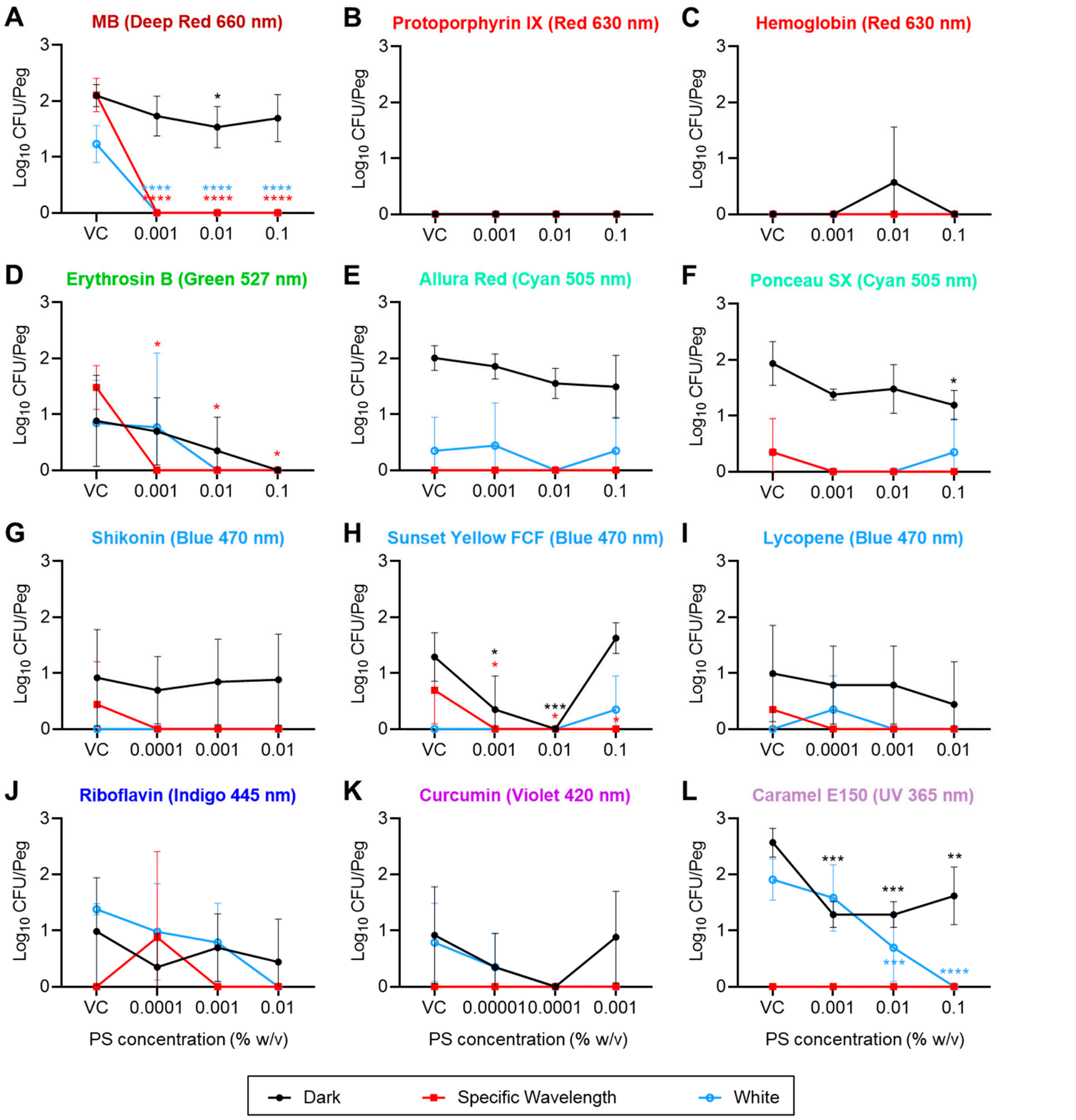

Figure S3. Recovery of viable *Brochothrix Thermosphacta* ATCC 11509 from MBEC pegs (Log$_{10}$ CFU/peg). Biofilms were challenged with methylene blue **(A)**, protoporphyrin IX **(B)**, hemoglobin **(C)**, erythrosin B **(D)**, allura red **(E)**, ponceau SX **(F)**, shikonin **(G)**, sunset yellow FCF **(H)**, tomato lycopene extract **(I)**, riboflavin **(J)**, curcumin **(K)**, or caramel color E150 **(L)**, at the indicated concentrations (x-axis; VC = Vehicle Controls), and subjected to the indicated irradiation conditions: dark (represented by black lines) vs specific wavelength (monochromatic light indicated above each graph, represented by red lines) vs white light (polychromatic light represented by blue lines). Symbols and error bars represent the mean ± standard deviation for 3 replicate pegs. Statistical significance, as indicated for certain data points, was evaluated by 2-way ANOVA and *p*-values (relative to the corresponding "VC" data point for each irradiation condition) were corrected for multiple comparisons using Dunnett's method. *, $p<0.05$; **, $p<0.01$; ***, $p<0.001$; ****, $p<0.0001$.

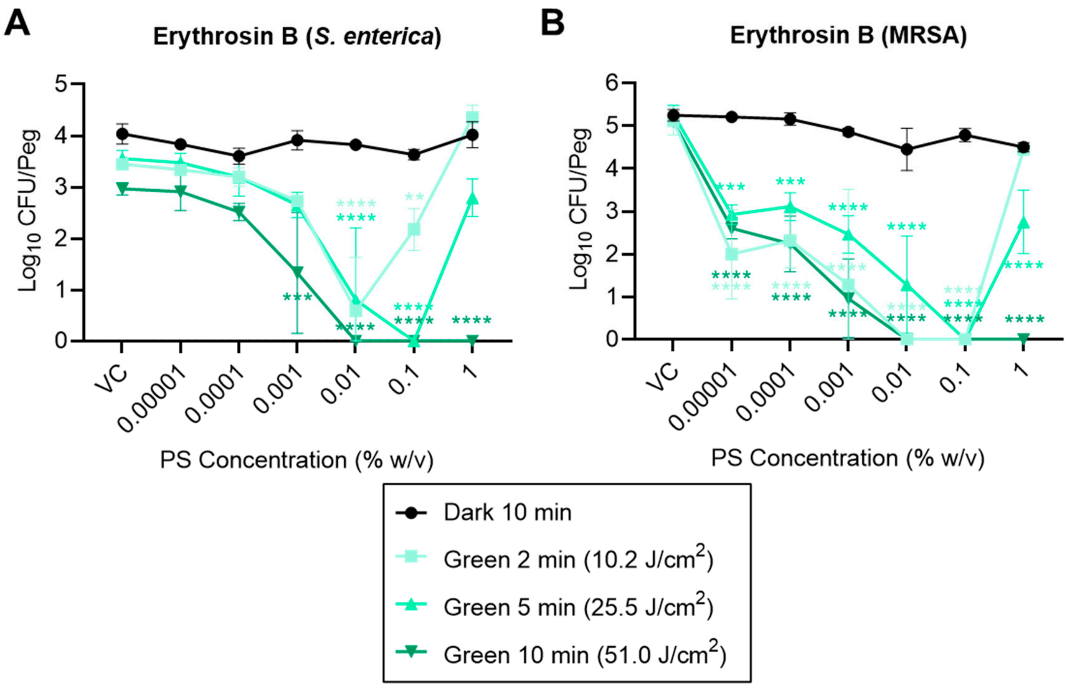

Figure S4. Recovery of viable *S. enterica* Choleraesuis ATCC 10708 (A) or methicillin resistant *Staphylococcus aureus* (MRSA 456 S. Yanke) (B) from green light-irradiated MBEC pegs (Log_{10} CFU/peg). Biofilms were challenged with erythrosin B, at the indicated concentrations (x-axis; VC = Vehicle Controls), for the indicated irradiation times. Symbols and error bars represent the mean ± standard deviation for 3 replicate pegs. Statistical significance, as indicated for certain data points, was evaluated by 2-way ANOVA and *p*-values (relative to the corresponding "VC" data point for each irradiation condition) were corrected for multiple comparisons using Dunnett's method. *, $p<0.05$; **, $p<0.01$; ***, $p<0.001$; ****, $p<0.0001$.

Table S1. Concentration of photosensitizer candidates used for screening.

Photosensitizer Candidate	Final Tested Concentrations (% w/v)	Final Tested Concentrations (mM)
**Methylene Blue (Positive Control)**	0.1, 0.01, 0.001	3.126, 0.313, 0.031
**Protoporphyrin IX**	0.1, 0.01, 0.001	1.777, 0.178,0.018
**Hemin**	0.01, 0.001, 0.0001	0.153, 0.0153, 0.0015
**Hemoglobin**	0.1, 0.01, 0.001	0.0155, 0.0016, 0.0002
**Titanium Dioxide**[e]	0.1, 0.01, 0.001	12.521, 1.252, 0.125
**Orchil (Orcein)**	0.01, 0.001, 0.0001	0.199, 0.019, 0.0019
**Cochineal (Carmine)**	0.01, 0.001, 0.0001	0.203, 0.0203, 0.002
**Anthocyanin (Cyanidin-3-glucoside)**	0.01, 0.001, 0.0001	0.206, 0.021, 0.0021
**Betanin (Beet Red)**	0.1, 0.01, 0.001	1.816, 0.182, 0.0018
**Black Carrot Extract**	0.1, 0.01, 0.001	-
**Erythrosin B**	0.1, 0.01, 0.001	1.136, 0.114, 0.0114
**Acid Red 27 (Amaranth)**	0.1, 0.01, 0.001	1.654, 0.165, 0.00165
**Enocyanin (an Anthocyanin)**	0.1, 0.01, 0.001	4.825, 0.482, 0.048
**Resveratrol (Grape Skin Extract)**	0.01, 0.001, 0.0001	0.438, 0.044, 0.0044
**Citrus red No. 2**	0.01, 0.001, 0.0001	0.324, 0.0324, 0.00324
**Allura Red**	0.1, 0.01, 0.001	2.014, 0.201, 0.0201
**Ponceau SX**	0.1, 0.01, 0.001	2.082, 0.208, 0.021
**Canthaxanthin**	0.001, 0.0001, 0.00001	0.018, 0.0018, 0.0002
**Shikonin (Alkanet)**	0.01, 0.001, 0.0001	0.347, 0.0347, 0.00347
**Sunset Yellow FCF**	0.1, 0.01, 0.001	2.2104, 0.221, 0.0221
**Tomato Lycopene Extract**	0.01, 0.001, 0.0001	0.186, 0.0186, 0.0019
**Paprika (Capsanthin)**	0.01, 0.001, 0.0001	0.038, 0.0038, 0.00038
**Trans-β-apo-8'-carotenal**	0.001, 0.0001, 0.00001	0.024, 0.0024, 0.00024
**Saffron**	0.1, 0.01, 0.001	1.024, 0.1024, 0.0102
**Xanthophyll (Lutein)**	0.01, 0.001, 0.0001	0.176, 0.0176, 0.0018
**Ethyl β-apo-8′-carotenoate**	0.001, 0.0001, 0.00001	0.022, 0.0022, 0.0022
**Riboflavin**	0.01, 0.001, 0.0001	0.266, 0.0266, 0.0027
**Curcumin**	0.001, 0.0001, 0.00001	0.027, 0.0027, 0.00027
**Beta-Carotene**	0.001, 0.0001, 0.00001	0.0186, 0.00186, 0.00019
**Tartrazine (Acid Yellow 23)**	0.1, 0.01, 0.001	1.871, 0.1871, 0.0187
**Norbixin (Annatto)**	0.01, 0.001, 0.0001	0.263, 0.0263, 0.0026
**Turmeric Extract**	0.001, 0.0001, 0.00001	-
**Caramel E150**	0.1, 0.01, 0.001	7.927, 0.793, 0.0793

Table S2. Treatment conditions used for the optimization of the photosensitizer candidates. Treatment conditions used for food contact surfaces including aluminum, stainless steel, galvanized steel, mild steel, copper, and wood.

PS	λ (nm)	Concentration Range (% w/v)	LED Irradiation Time	Dose (J/cm^2)
**Treatment conditions for optimization of PS candidates**				
**Riboflavin-5'-Phosphate Sodium USP**	470 (Blue)	1<br>0.1<br>0.01<br>0.001<br>0.0001<br>0.00001<br>VC=water	5.0 min<br>1.0 min<br>2.5 min<br>5.0 min	0<br>10.2<br>25.5<br>51.0
**Sunset Yellow**		1<br>0.1<br>0.01<br>0.001<br>0.0001<br>0.00001<br>VC=water		
**Curcumin**		0.001<br>0.0001<br>0.00001<br>0.000001<br>0.0000001<br>0.00000001<br>VC=1% DMSO in water		
**Erythrosin B**	527 (Green)	1<br>0.1<br>0.01<br>0.001<br>0.0001<br>0.00001<br>VC=water	10.0 min<br>2.0 min<br>5.0 min<br>10.0 min	
**Treatment conditions for food contact surfaces**				
**Riboflavin-5'-Phosphate**	445 nm (Indigo)	0.1	5 min	55.5

Table S3. The minimum bactericidal concentration (MBC) and minimum biofilm eradication concentration (MBEC) values (in % w/v) of the less effective photosensitizers (based on the turbidimetric assay for *Salmonella enterica* Choleraesuis ATCC 10708, Methicillin Resistant *Staphylococcus aureus* (MRSA 456 S. Yanke), *Pseudomonas fragi* (ATCC 4973), and *Brochothrix Thermosphacta* (ATCC 11509).

Photosensitizer	LED	Turbidimetric Assay							
		S. enterica		**MRSA**		***P. fragi***		***B. thermosphacta***	
		**MBC**	**MBEC**	**MBC**	**MBEC**	**MBC**	**MBEC**	**MBC**	**MBEC**
**Protoporphyrin IX**	Dark	>0.1	>0.1	>0.1	>0.1	>0.1	>0.1	N/A	N/A
	Red	>0.1	>0.1	>0.1	>0.1	>0.1	0.1?	N/A	N/A
	White	0.1?	>0.1	0.01	0.01	0.1	0.1	N/A	N/A
**Hemin**	Dark	>0.01	>0.01	>0.01	>0.01	>0.01	>0.01	N/A	N/A
	Red	>0.01	>0.01	>0.01	>0.01	>0.01	>0.01	N/A	N/A
	White	>0.01	>0.01	>0.01	>0.01	>0.01	>0.01	N/A	N/A
**Titanium dioxide**	Dark	>0.1	>0.1	>0.1	>0.1	>0.1	>0.1	>0.1	>0.1
	Amber	>0.1	>0.1	>0.1	>0.1	>0.1	>0.1	>0.1	>0.1
	White	>0.1	>0.1	>0.1	>0.1	>0.1	>0.1	>0.1	>0.1
**Orcein (Orchil)**	Dark	>0.01	>0.01	>0.01	>0.01	>0.01	>0.01	>0.01	>0.01
	Amber	>0.01	>0.01	>0.01	>0.01	>0.01	>0.01	>0.01	>0.01
	White	>0.01	>0.01	>0.01	>0.01	>0.01	>0.01	0.01?	0.01?
**Carmine (Cochineal)**	Dark	>0.01	>0.01	>0.01	>0.01	>0.01	>0.01	>0.01	>0.01
	Amber	>0.01	>0.01	>0.01	>0.01	>0.01	>0.01	0.01	0.001
	White	>0.01	>0.01	>0.01	>0.01	>0.01	>0.01	>0.01	>0.01
**Cyanidin-3-O-glucoside**	Dark	>0.01	>0.01	>0.01	>0.01	>0.01	>0.01	>0.01	>0.01
	Green	>0.01	>0.01	>0.01	>0.01	>0.01	>0.01	>0.01	>0.01
	White	>0.01	>0.01	>0.01	>0.01	>0.01	>0.01	>0.01	>0.01
**Betanin**	Dark	>0.1	>0.1	>0.1	>0.1	>0.1	>0.1	>0.1	>0.1
	Green	>0.1	>0.1	>0.1	>0.1	>0.1	>0.1	>0.1	>0.1
	White	>0.1	>0.1	>0.1	>0.1	>0.1	>0.1	>0.1	>0.1
**Black Carrot Extract**	Dark	>0.1	>0.1	>0.1	>0.1	>0.1	>0.1	>0.1	>0.1
	Green	>0.1	>0.1	>0.1	>0.1	>0.1	>0.1	>0.1	>0.1
	White	>0.1	>0.1	>0.1	>0.1	>0.1	>0.1	>0.1	>0.1
**Acid Red 27 (Amaranth)**	Dark	>0.1	>0.1	>0.1	>0.1	>0.1	>0.1	>0.1	>0.1
	Green	>0.1	>0.1	>0.1	>0.1	>0.1	>0.1	0.01	0.01
	White	0.1?	0.01?	>0.1	>0.1	0.1?	0.01	0.01	0.01
**Enocyanin**	Dark	>0.1	>0.1	>0.1	>0.1	>0.1	>0.1	0.1?	>0.1
	Green	>0.1	>0.1	>0.1	>0.1	>0.1	>0.1	0.1	0.1?
	White	>0.1	>0.1	>0.1	>0.1	>0.1	>0.1	0.1	0.1?
**Resveratrol**	Dark	>0.01	>0.01	>0.01	>0.01	>0.01	>0.01	>0.01	>0.01
	Green	>0.01	>0.01	>0.01	>0.01	>0.01	>0.01	>0.01	>0.01
	White	>0.01	>0.01	>0.01	>0.01	>0.01	>0.01	>0.01	>0.01
**Citrus Red No. 2**	Dark	>0.01	>0.01	>0.01	>0.01	>0.01	>0.01	>0.01	>0.01
	Cyan	>0.01	>0.01	>0.01	>0.01	>0.01	>0.01	>0.01	N/A
	White	>0.01	>0.01	>0.01	>0.01	>0.01	>0.01	>0.01	>0.01
**Canthaxanthin**	Dark	>0.01	>0.01	>0.01	>0.01	>0.001	>0.001	>0.001	>0.001
	Blue	>0.01	>0.01	>0.01	>0.01	>0.001	>0.001	>0.001	>0.001
	White	>0.01	>0.01	>0.01	>0.01	>0.001	>0.001	N/A	>0.001
**Capsanthin**	Dark	>0.01	>0.01	>0.01	>0.01	>0.01	>0.01	>0.01	>0.01
	Blue	>0.01	>0.01	>0.01	>0.01	>0.01	>0.01	0.01?	N/A
	White	>0.01	>0.01	>0.01	>0.01	>0.01	>0.01	>0.01	N/A

**Trans-β-apo-8'-carotenal**	Dark	>0.001	>0.001	>0.001	>0.001	>0.001	>0.001	>0.001	>0.001
	Blue	>0.001	>0.001	>0.001	>0.001	>0.001	>0.001	0.0001?	N/A
	White	>0.001	>0.001	>0.001	>0.001	>0.001	>0.001	>0.001	0.001?
**Saffron**	Dark	>0.1	>0.1	>0.1	>0.1	>0.1	>0.1	>0.1	>0.1
	Blue	>0.1	>0.1	>0.1	>0.1	0.1?	0.1	0.1?	N/A
	White	>0.1	>0.1	>0.1	>0.1	>0.1	>0.1	>0.1	0.01
**Lutein**	Dark	>0.01	>0.01	>0.01	>0.01	>0.01	>0.01	>0.01	>0.01
	Indigo	>0.01	>0.01	>0.01	>0.01	>0.01	>0.01	>0.01	N/A
	White	>0.01	>0.01	>0.01	>0.01	>0.01	>0.01	>0.01	>0.01
**Ethyl β-apo-8'-carotenoate**	Dark	>0.001	>0.001	>0.001	>0.001	>0.001	>0.001	>0.001	>0.001
	Indigo	>0.001	>0.001	>0.001	>0.001	>0.001	>0.001	>0.001	N/A
	White	>0.001	>0.001	>0.001	>0.001	>0.001	>0.001	>0.001	>0.001
**β-carotene**	Dark	>0.001	>0.001	>0.001	>0.001	>0.001	>0.001	0.001?	0.001
	Violet	0.0001?	0.0001	>0.001	>0.001	>0.002	N/A	N/A	0.001?
	White	>0.001	>0.001	>0.001	>0.001	>0.001	>0.001	>0.001	N/A
**Acid Yellow 23**	Dark	>0.1	>0.1	>0.1	>0.1	>0.1	>0.1	0.1?	N/A
	Violet	0.001*	N/A	0.01	0.01	0.001?	N/A	N/A	0.1
	White	>0.1	>0.1	>0.1	>0.1	>0.1	>0.1	>0.1	N/A
**Norbixin**	Dark	>0.01	>0.01	0.01	>0.01	>0.01	>0.01	0.01	N/A
	Violet	0.001*	0.001*	0.01	0.001	0.001	N/A	N/A	N/A
	White	>0.01	>0.01	>0.01	>0.01	>0.01	>0.01	0.01	N/A
**Turmeric extract**	Dark	>0.001	>0.001	>0.001	>0.001	>0.001	>0.001	0.001?	0.001?
	Violet	N/A	0.00001*	>0.001	>0.001	0.001?	N/A	N/A	>0.001
	White	>0.001	>0.001	>0.001	>0.001	>0.001	>0.001	>0.001	N/A

? = 2 of 3 wells clear
N/A = No growth in at least 2 of 3 VC wells
* = only 2 of 3 VC wells grew
Max = "Maximum kill" (zero CFUs recovered from the treated wells; magnitude of the log reduction is determined by the recovery from the VC wells).
For the quantitative data, green highlighting indicates p values <0.05 (based on an unpaired, two-tailed T-test, relative to the appropriate vehicle control) or $\log_{10}$ CFU/peg reductions >1.

Table S4. The minimum bactericidal concentration (MBC) and minimum biofilm eradication concentration (MBEC) values (in % w/v) of riboflavin and riboflavin-5'-phosphate (based on the turbidimetric and quantitative assay) for MRSA and *Salmonella enterica* Choleraesuis (ATCC 10708). Recovery of viable MRSA and *S. enterica* from indigo irradiated MBEC pegs (Log_{10} CFU/peg) are also included. Biofilms were challenged with riboflavin and riboflavin-5'-phosphate at the indicated concentrations, for 5 min (corresponding to 55.5 J/cm^2 dose) with 445 nm LED light (n=3).

PS	Organism	Turbidimetric Assay		Quantitative assay	
		MBC (% w/v)	MBEC (% w/v)	Quant. MBEC (% w/v)	Log_{10} CFU reduction[a]
**Riboflavin**	MRSA	>0.01	>0.01	>0.01	1.76±0.43
	S. enterica	>0.01	>0.01	>0.01	1.04±1.31
**Riboflavin-5-Phosphate**	MRSA	0.01	0.01	0.01	4.17^{max}±0.00
	S. enterica	0.1	0.1	0.1	3.05^{max}±0.00

[a] Values in this column are given as mean ± SD (n=3).

Table S5. The minimum bactericidal concentration (MBC) and minimum biofilm eradication concentration (MBEC) values (in % w/v) of the selected photosensitizers (based on the turbidimetric and quantitative assay) for *Salmonella enterica* Choleraesuis (ATCC 10708).

PS	Dose (J/cm^2)	Turbidimetric Assay		Quantitative Assay	Notes
		MBC (% w/v)	MBEC (% w/v)	Quant. MBEC (% w/v)	
**Riboflavin-5'-Phosphate**	0 (Dark)	>1	>1	>1	
	10.2	>1	>1	>1	
	25.5	>1	>1	>1	
	51.0	0.1	0.01	0.1	
**Sunset Yellow**	0 (Dark)	>1	>1	>1	
	10.2	>1	>1	>1	
	25.5	>1	>1	>1	
	51.0	0.01	0.001	0.001	
**Curcumin**	0 (Dark)	>0.001	>0.001	>0.001	
	10.2	>0.001	>0.001	>0.001	
	25.5	>0.001	>0.001	>0.001	
	51.0	>0.001	>0.001	0.0001	
**Riboflavin-5'-Phosphate + Sunset Yellow**	0 (Dark)	>1, >1	>1, >1	>1, >1	**Antagonistic effect**: Sunset Yellow lost potency relative to individual.
	10.2	>1, >1	>1, >1	>1, >1	
	25.5	>1, >1	>1, >1	>1, >1	
	51.0	0.01, 0.01	0.01, 0.01	0.01, 0.01	
**Sunset Yellow + Curcumin**	0 (Dark)	>1, >0.001	>1, >0.001	>1, >0.001	**Neutral**: Sunset Yellow same potency as individual; Curcumin did not hinder.
	10.2	>1, >0.001	>1, >0.001	>1, >0.001	
	25.5	>1, >0.001	>1, >0.001	>1, >0.001	
	51.0	0.001, 0.000001	0.001, 0.000001	0.001, 0.000001	
**Riboflavin-5'-Phosphate + Curcumin**	0 (Dark)	>1, >0.001	>1, >0.001	>1, >0.001	**Synergistic effect**: Improved efficacy at 2.5 min = synergy. Improved efficacy at 0.01% R-5-P, 0.00001% Curcumin = Synergy.
	10.2	>1, >0.001	>1, >0.001	>1, >0.001	
	25.5	0.1, 0.0001	0.1, 0.0001	0.1, 0.0001	
	51.0	0.1, 0.0001	0.1, 0.0001	0.01, 0.00001	
**Erythrosin B**	0 (Dark)	>1	>1	>1	Erythrosin B is potent, even at 25.5 J/cm^2.
	10.2	0.01	0.01	>1	
	25.5	0.01	0.01	0.1	
	51.0	0.01	0.01	0.01	

Note: For *S. enterica*, "Quant. MBEC" = lowest concentration yielding either >3 $\log_{10}$ reduction OR max kill (whichever is greater). For turbidimetric data, yellow shading indicates that 3 of 4 wells were clear ("Blue" set) or 2 of 3 wells were clear (Erythrosin B).

Table S6. The minimum bactericidal concentration (MBC) and minimum biofilm eradication concentration (MBEC) values (in % w/v) of the selected photosensitizers (based on the turbidimetric and quantitative assay) for Methicillin resistant *Staphylococcus aureus* (MRSA 456 S. Yanke).

PS	Dose (J/cm^2)	Turbidimetric Assay		Quantitative Assay	Notes
		MBC (% w/v)	MBEC (% w/v)	Quant. MBEC (% w/v)	
**Riboflavin-5'-Phosphate**	0 (Dark)	>1	>1	>1	
	10.2	>1	>1	>1	
	25.5	0.1	>1	>1	
	51.0	0.1	0.1	0.1	
**Sunset Yellow**	0 (Dark)	>1	>1	>1	
	10.2	>1	>1	>1	
	25.5	>1	>1	>1	
	51.0	0.01	0.01	0.01	
**Curcumin**	0 (Dark)	>0.001	>0.001	>0.001	
	10.2	>0.001	>0.001	>0.001	
	25.5	>0.001	>0.001	>0.001	
	51.0	0.001	>0.001	0.001	
**Riboflavin-5'-Phosphate + Sunset Yellow**	0 (Dark)	>1, >1	>1, >1	>1, >1	**Neutral**: Sunset Yellow same potency as individual; R-5-P did not hinder.
	10.2	>1, >1	>1, >1	>1, >1	
	25.5	>1, >1	>1, >1	>1, >1	
	51.0	0.01, 0.01	0.01, 0.01	0.01, 0.01	
**Sunset Yellow + Curcumin**	0 (Dark)	>1, >0.001	>1, >0.001	>1, >0.001	**Neutral**: Sunset Yellow same potency as individual; Curcumin did not hinder.
	10.2	>1, >0.001	>1, >0.001	>1, >0.001	
	25.5	>1, >0.001	>1, >0.001	>1, >0.001	
	51.0	0.01, 0.00001	0.01, 0.00001	0.01, 0.00001	
**Riboflavin-5'-Phosphate + Curcumin**	0 (Dark)	>1, >0.001	>1, >0.001	>1, >0.001	**Neutral**: R-5-P same potency as individual; Curcumin did not hinder.
	10.2	0.1, 0.0001	>1, >0.001	>1, >0.001	
	25.5	0.1, 0.0001	>1, >0.001	>1, >0.001	
	51.0	0.1, 0.0001	0.1, 0.0001	0.1, 0.0001	
**Erythrosin B**	0 (Dark)	1	>1	>1	Erythrosin B is potent, even at 10.2 J/cm^2.
	10.2	0.001	0.1	0.01	
	25.5	0.0001	0.1	0.01	
	51.0	0.0001	0.01	0.001	

Note: For MRSA, "Quant. MBEC" = lowest concentration yielding >4 log_{10} reduction. For turbidimetric data, yellow shading indicates that 3 of 4 wells were clear ("Blue" set).
